# Air-Gap Interrogation of Surface Plasmon Resonance in Otto Configuration

**DOI:** 10.3390/mi12080998

**Published:** 2021-08-21

**Authors:** Yeonsu Lee, Jiwon Kim, Sungmin Sim, Ignacio Llamas-Garro, Jungmu Kim

**Affiliations:** 1 School of Electronic and Information Engineering, Jeonbuk National University, Jeonju 54896, Korea; ys1531.lee@samsung.com (Y.L.); inpachens@jbnu.ac.kr (J.K.); 2 Korea Institute of Industrial Technology, Ansan 15588, Korea; ssm2262@kitech.re.kr; 3 Centre Tecnològic de Telecomunicacions de Catalunya, CTTC/CERCA, 08860 Castelldefels, Spain; ignacio.llamas@cttc.es

**Keywords:** surface plasmon resonance, Kretschmann configuration, Otto configuration, piezoactuator, MEMS actuator, FEM simulation

## Abstract

In this study, a micromachined chip in Otto configuration with multiple air-gaps (1.86 μm, 2.42 μm, 3.01 μm, 3.43 μm) was fabricated, and the resonance characteristics for each air-gap was measured with a 980 nm laser source. To verify the variability of the reflectance characteristics of the Otto configuration and its applicability to multiple gas detection, the air-gap between the prism and metal film was adjusted by using a commercial piezoactuator. We experimentally verified that the SPR characteristics of the Otto chip configuration have a dependence on the air-gap distance and wavelength of the incident light. When a light source having a wavelength of 977 nm is used, the minimum reflectance becomes 0.22 when the displacement of the piezoactuator is about 9.3 μm.

## 1. Introduction

Surface plasmon polaritons are collective oscillations of electrons on the surface of the metals such as gold, silver, and aluminum, and it is caused by an incident light. When specific conditions such as the light source angle of incidence, wavelength, and refractive index of materials are satisfied, the incident light can be absorbed as surface plasmon polaritons, and the reflectance is rapidly decreased. This phenomenon is called the surface plasmon resonance (SPR). Surface plasmon resonance characteristics are changed sensitively according to a dielectric constant and refractive index change of the materials near the metal surface. Thus, if target molecules enter in contact with the metal surface, the surface plasmon resonance effect will take place, and its degree of excitation is used to make sensor systems. Through the last decade [1,2,3,4,5], SPR sensors have been widely used for chemical gas detection, drug design, and biochemical reaction detection with the advantages of label-free and room temperature detection, with real-time monitoring. Recently, SPR characteristics has been applied as a sensor system to detect physical parameters, such as micro-displacement and pressure change [6,7,8]. There are two types of coupling configurations for surface plasmon resonance system using thin metal films, namely the Kretschmann and Otto configurations [9,10].

**Figure 1:**
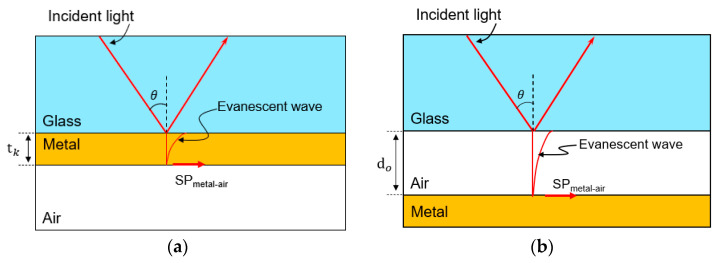
Prism coupling configurations used in SPR systems: (**a**) Kretschmann configuration; (**b**) Otto configuration.

Figure 1a shows the Kretschmann configuration composed of a thin metal film and a glass substrate [9]. Since the refractive index of the glass is higher compared to the metal film, an incident light is totally reflected at the interface between the glass substrate and the thin metal film. At this time, an evanescent wave can propagate along the interface of the two materials. Generally, the magnitude of the evanescent wave decreases exponentially inside of the metallic medium. If the metal film thickness ($t_{k}$) is thin enough for the wavelength of the incident light, the end of the evanescent wave can excite the surface plasmon (SP_metal-air_) at the bottom side of the metal film. Therefore, one of the most important parameters related to the Kretschmann configuration-based SPR is the thickness of the metal film. In case of the Otto configuration-based SPR sensors, it is necessary that the glass and metal film have an air-gap distance of about sub-micron ($d_{o})$ sizes, which is a key parameter, as shown in Figure 1b [10,11]. Due to the robust structure and convenience of fabrication, most of the surface plasmon resonance sensors are based on the Kretschmann configuration. However, once the Kretschmann configuration-based SPR sensor had been fabricated, it is difficult to realize a real-time adjustment of the metal thickness. By contrast, the Otto configuration has the potential of reconfigurability, where tunable resonance characteristics are obtained according to a variable air-gap distance. If the air-gap between the glass substrate and the thin metal film can be stably adjusted, it will be possible to use a surface plasmon resonance sensor with the structural flexibility obtained using the Otto configuration. This feature implies that the Otto configuration-based SPR sensor has potential application in multiple gas detection. A comparison between the Otto and Kretschmann configuration can be found in [12]. Table 1 shows the characteristics of the Kretschmann and Otto configurations.

Many researchers focused on the application of the Otto configuration and concluded their research by using theoretical simulations without experimental results [13,14,15,16]. According to the development of micromachining technology, the Otto configuration-based SPR sensor systems have been experimentally realized [17,18]. Sopko et al. proposed the concept of an acousto-optical device using Otto configuration-based SPR phenomenon with 10.6 μm wavelength [18,19]. Although their sensor system can be used to analyze dependencies of the reflective coefficient of the prism–air–metal system on the air-gap and dielectric permittivity, a relationship between the air-gap and resonance wavelength is not referred. Moreover, most of the SPR systems that use the prism coupling configuration operated with an angular interrogation method having a large size because of the rotation stage and motor driver used [20]. 

**Figure 2:**
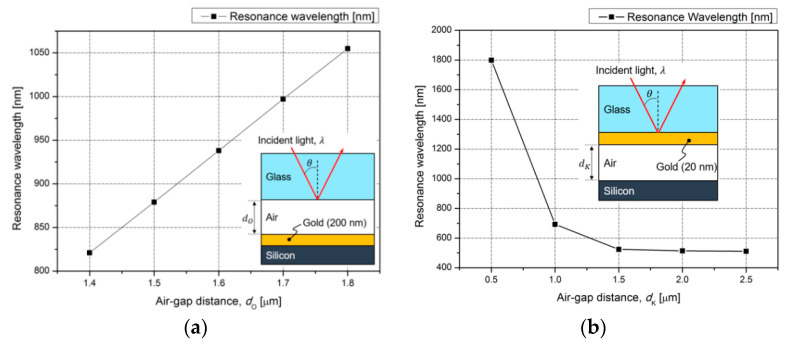
SPR resonance wavelength according to the air-gap distance: (**a**) Otto configuration; (**b**) Kretschmann configuration.

Figure 2 shows the dependence of the resonance wavelength on the air-gap for Kretschmann and Otto configurations where the two systems are composed of glass, metal, and silicon substrate. In case of the Kretschmann configuration, the resonance wavelength is converged to the specific value according to the increasing air-gap distance between the metal film and silicon substrate, as shown in Figure 2a. This tendency can be explained, since the resonance wavelength is determined by the thickness of the metal film and can be easily affected by the silicon in the proximity of the surface plasmon polariton. The resonance wavelength of the Otto configuration is influenced by the air-gap distance, as shown in Figure 2b. It should be noticed that the resonance wavelength can be linearly adjusted by increasing the air-gap distance. As a result, the Otto configuration has potential use for an SPR system using the air-gap interrogation method, which in this work was achieved using a micro-actuator.

In this study, we verified the feasibility of an Otto configuration-based SPR sensor system using the air-gap interrogation method. In Section 2, we propose the Otto configuration with variable air-gaps including measurement results of the SPR characteristics and a comparison with the FEM simulation results. Section 3 explains the feasibility of the air-gap interrogation method for the Otto SPR system. The Otto configuration SPR characteristics dependance on the air-gap distance is analyzed by using a proposed SPR measurement system including a piezoactuator. 

## 2. Otto Configuration with Multiple Air-Gaps

### 2.1. Design

**Figure 3:**
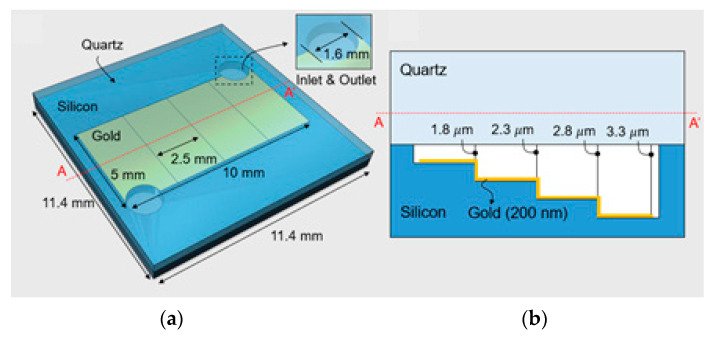
Otto configuration-based SPR sensor chip with stepped air-gap height: (**a**) schematic view; (**b**) cross-section view.

Figure 3a shows a schematic view of the proposed Otto configuration with multiple air-gaps. The SPR chip is composed of a silicon substrate with a quartz cap. The silicon has a step-like cavity, which is separated into four sections. Taking into account the wavelength of 980 nm, a 200 nm-thick gold film is located in the cavity, and the air-gaps between the metal film and quartz cap are designed to be 1.8 μm, 2.3 μm, 2.8 μm, and 3.3 μm, respectively, as shown in Figure 3b. Each section of the silicon cavity has the same width of 2.5 mm according to the beam diameter of the laser source. At both ends of the silicon cavity, an inlet and outlet with a diameter of 1.6 mm for gas flow is integrated on the chip to conduct further studies. The whole size of the proposed SPR chip is 11.4 × 11.4 × 1 mm^3^. 

### 2.2. Fabrication

**Figure 4:**
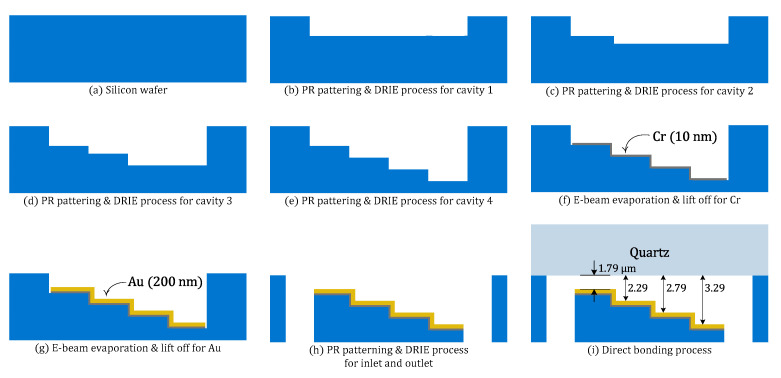
SPR sensor chip fabrication process with stepped air-gaps (**a**) silicon substrate; (**b**) first DRIE process; (**c**) second DRIE process; (**d**) third DRIE process; (**e**) fourth DRIE process; (**f**) Cr evaporation; (**g**) Au evaporation; (**h**) DRIE for inlet and outlet; (**i**) direct bonding process.

The proposed sensor chip is fabricated following the process shown in Figure 4. Firstly, a cavity with four different depths of 2 μm, 2.5 μm, 3 μm, and 3.5 μm is formed at the surface of a 500 μm-thick, 4-inch silicon wafer though the deep reactive ion etching (DRIE) process where photoresist is used as an etching mask. Then, a 200 nm-thick gold layer is patterned inside the cavity with an adhesion layer of 10 nm-thick chrome by using an electron-beam (E-beam) evaporation and lift-off process. The inlet and outlet for fluids sensing test is defined through the backside using the DRIE process. Finally, the prepared silicon and a 500 μm-thick quartz substrate are bonded together to form the Otto chip. To assure that the two wafers have a clean surface, an isopropyl alcohol (IPA)-based solution and sulfuric acid-based SPM solution is used to remove any contamination on the wafers. After that, to make each wafer surface hydrophilic, the wafers are treated with O_2_ plasma (RF power: 150 W, pressure: 100 mTorr, O_2_ flow rate: 50 sccm, and treatment time: 10 s) by using an inductively coupled plasma (ICP) etcher system before the direct bonding process. The direct bonding process is completed with manual bonding and thermal treatment (temp: 300 °C, treatment time: 8 h). 

**Figure 5:**
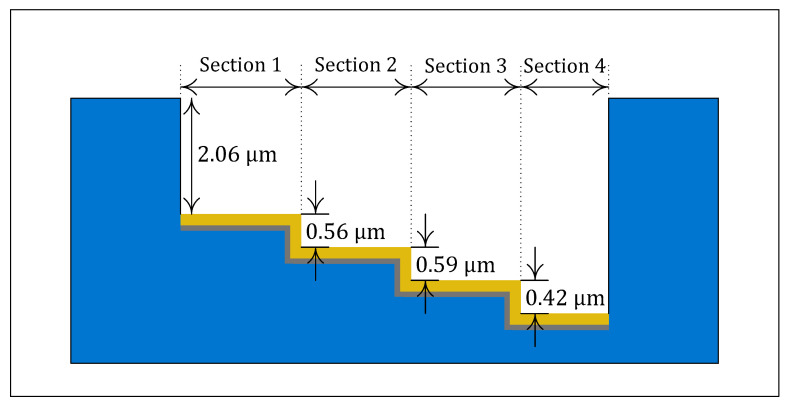
Measured profile of the silicon cavity after the first DRIE process.

**Figure 6:**
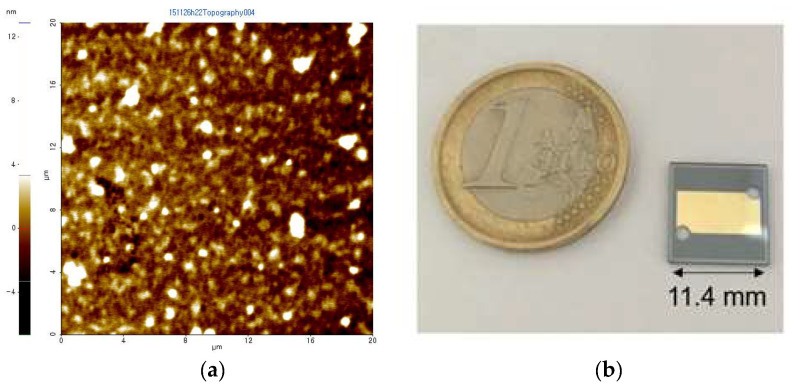
Fabrication results (**a**) surface roughness of the metal film; (**b**) SPR chip with multiple air-gaps.

Figure 5 shows the silicon cavity profile after the fourth DRIE process, as shown in Figure 4b without the gold and chrome film, which is measured using a profilometer (Alpha-step IQ, KLA-Tencor, Inc.). Each step height is measured to be about 2.06 μm, 0.56 μm, 0.59 μm, and 0.42 μm, respectively. Therefore, it is expected that the final fabricated SPR chip has air-gaps of about 1.86 μm, 2.42 μm, 3.01 μm, and 3.43 μm, respectively, where the sections are called Section 1, Section 2, Section 3, and Section 4, at each position. The surface roughness of the gold film is also measured to be about 1.3 nm by using an atomic force measurement (AFM) system (XE-100, Parksystems, Inc.), as shown in Figure 6a. The measured surface roughness level is too small to degrade the SPR characteristics of the sensor chip. The fabricated Otto configuration-based SPR chip with multiple air-gaps is shown in Figure 6b.

### 2.3. Measurement and Results

**Figure 7:**
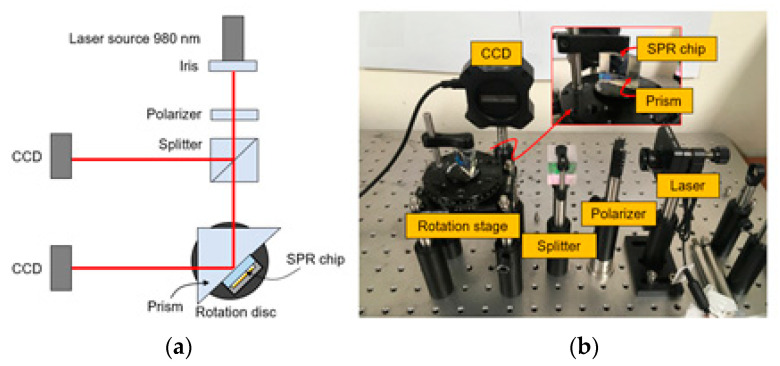
SPR measurement setup (**a**) schematic view; (**b**) measurement setup and fabricated SPR sensor chip.

SPR characteristics of the fabricated Otto configuration-based sensor chip is achieved by using the measurement setup shown in Figure 7. Before experimenting with the polychromatic module, the experiment is performed using a laser module. The laser module has a 980 nm wavelength used as incident light, and its beam diameter is controlled to be about 2 mm by using an optical iris. To excite the surface plasmon on the metal surface, the polarization of light should be parallel to the incidence plane of the measurement system. A TM polarized laser beam is incident on the BK7 prism, which is located on the rotation stage, and the fabricated SPR chip is attached on the hypotenuse side of the prism by using an index-matching oil. The use of the index-matching oil plays a vital role, preventing the degradation of SPR characteristics caused by unintended scattered reflection at the minute gap between the prism and SPR chip. The optical intensity of the reflected light at the interface between the glass substrate and air-gap of the fabricated SPR chip is measured using a CCD camera. In addition, part of the incident light is measured as a reference signal, and the incidence angle can be controlled by the rotation stage. The measurements are taken in two steps. First, the position of the desired chip section is selected by adjusting the height of the sample vertically. Then, the incident angle is adjusted with the dial located on the rotation stage. The dial changes the incident angle by 0.25 degrees.

**Figure 8:**
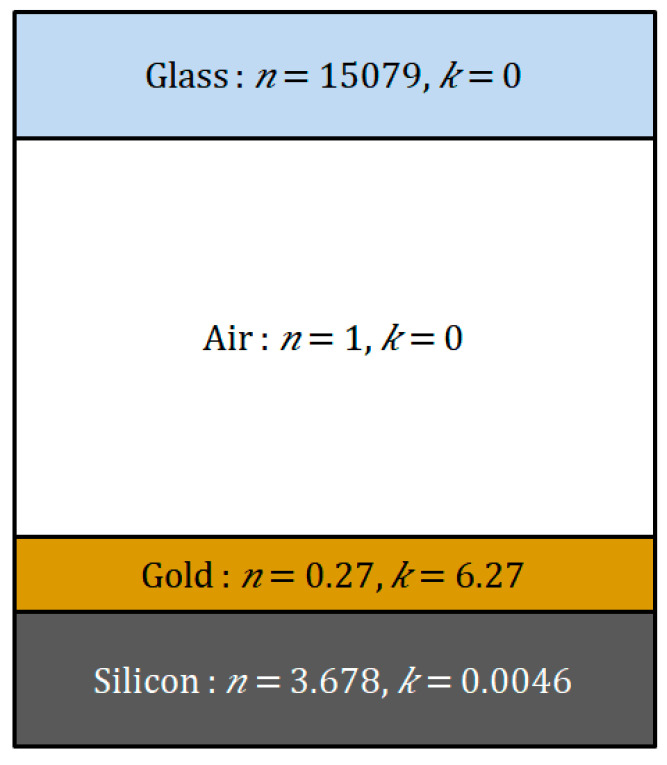
Schematic of the design used in simulations with corresponding refractive indexes.

**Figure 9:**
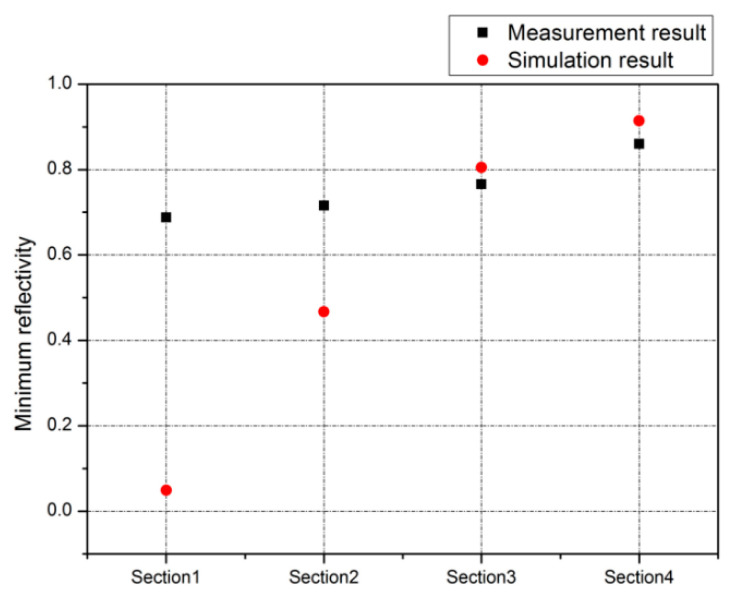
Measurement result of the fabricated SPR sensor chip.

The simulation schematic used to produce the design is shown in Figure 8; simulation and measurement comparison is shown in Figure 9. As depicted in Figure 9, minimum reflectance when the SPR phenomenon occurred is measured to be 0.688, 0.716, 0.766, and 0.86, respectively at each chip section; the measurement results are compared with theoretically calculated results by using FEM simulation (COMSOL Multiphysics, Altsoft Inc.). The resonance angles at each chip section are 42.88°, 42.48°, 41.77°, and 44.44°, respectively. The minimum reflectance is minimized at Section 1, and the reflectance is increased according to the increasing air-gap distance. This means that the SPR phenomenon is maximized at Section 1, and the Otto configuration-based SPR characteristics can be affected by the air-gap distance. 

## 3. Air-Gap Interrogation of Otto SPR System Using a Piezoactuator

### 3.1. Otto Configuration-Based SPR System Using a Piezoactuator

**Figure 10:**
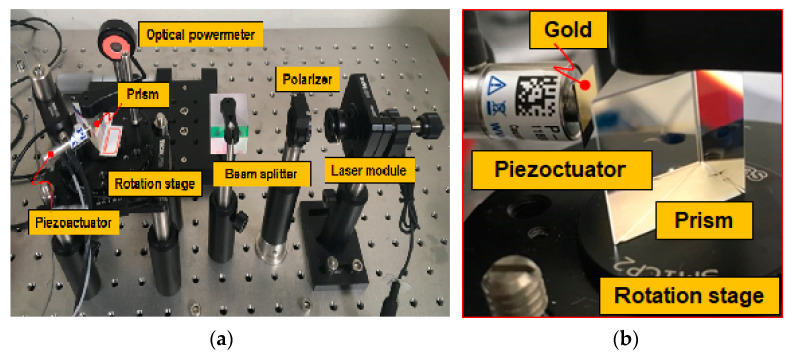

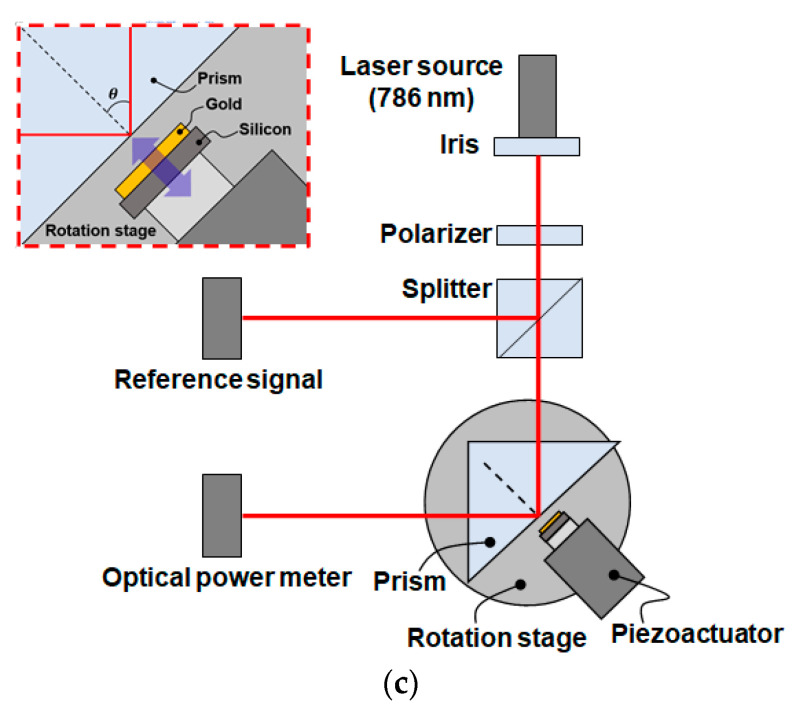
Reconfigurable Otto SPR system using a piezoactuator. (**a**) reconfigurable Otto SPR system experimental setup; (**b**) piezoactuator experimental setup; (**c**) schematic view.

In this section, we report the feasibility of an air-gap interrogation of the Otto chip configuration. To verify the characteristic variability of the Otto configuration, and its applicability, the air-gap between the prism and the metal film is adjusted by using a commercial piezoactuator (P-841.10, PI Korea Ltd., Seoul, Korea). When a 2mV_pp_ voltage is applied to the piezoactuator, the response behavior of the piezoactuator shows a 2 nm position change. Figure 10a shows the proposed Otto configuration-based SPR measurement system. In this experiment, we used laser modules with two different wavelengths of 786 nm and 977 nm. The power of the reflected light is measured by using an optical power meter (PM121D, Thorlabs Inc., Newton, MA, USA). To build the Otto configuration, a 200 nm-thick gold film is evaporated on the 500 nm-thick silicon die, which is attached to the moving head of the piezoactuator. Thus, it is easy to set up the Otto configuration-based SPR measurement system by aligning the piezoactuator and the prism without any complex fabrication process. In this system, the incident light is reflected at the hypotenuse side of the prism, and the air-gap distance between the gold film and prism can be adjusted by the piezoactuator with sub-micron resolution in real time. 

### 3.2. SPR Characteristics with Air-Gap Variation

**Figure 11:**
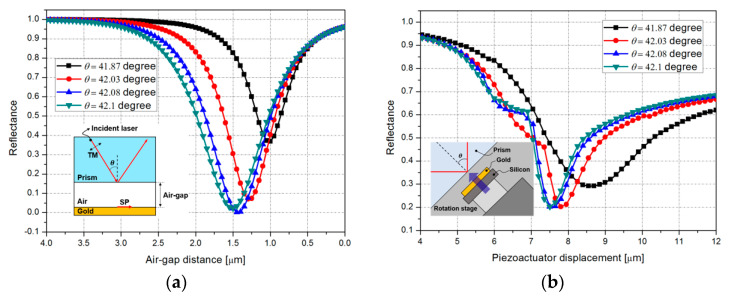
SPR characteristics with air-gap variation: (**a**) simulation result; (**b**) measurement result.

Figure 11a shows the FEM simulation result of the Otto configuration-based SPR characteristics according to the variable air-gap distance from 4 to 0 μm at a wavelength of 786 nm. In this case, the incident angle is assumed to be 41.87°, 42.03°, 42.08°, and 42.1°, each minimum reflectance is calculated to be 0.368, 0.07, 0.001, and 0.023, for each air-gap 1 μm, 1.3 μm, 1.45 μm, and 1.5 μm, respectively. This result means that the SPR phenomenon can be maximized when the air-gap and incident angle are 1.45 μm, 42.08° for the wavelength of 786 nm. As depicted in Figure 11b, we measured the reflectance according to the variable displacement of the piezoactuator, from 4 to 12 μm. The measured minimum reflectance values for the incident angles of 41.87°, 42.03°, 42.08°, and 42.1° are 0.292, 0.202, 0.202, and 0.202 where the displacement of the piezoactuator is 8.55 μm, 7.8 μm, 7.5 μm, and 7.5 μm, respectively. The measurement result shows that the maximum SPR phenomenon occurs when the air-gap and incident angle are 7.5 μm and 42.1°. Table 2 Shows FWHM of simulation result and measurement result.

### 3.3. SPR Characteristics with Air-Gap and Wavlength Variation

**Figure 12:**
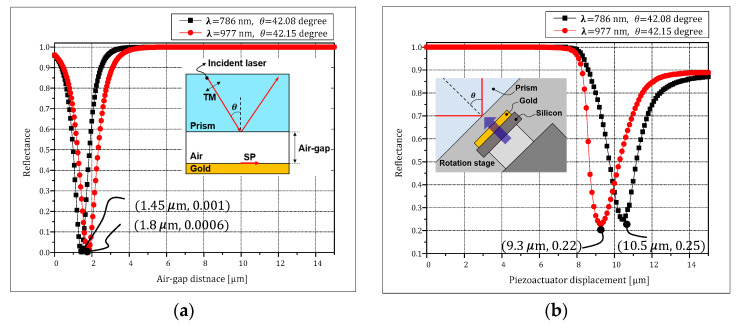
SPR characteristics according to air-gap and wavelength: (**a**) simulation result; (**b**) measurement result.

We experimentally verified that the SPR characteristics of the Otto configuration have a dependence on the air-gap distance and the wavelength of the incident light. Figure 12a shows a simulation result where the wavelengths are 786 nm and 977 nm, with incident angles of 42.08°and 42.15°, respectively. According to this simulation result, the minimum reflectance of about zero can be achieved when the air-gap is 1.45 μm and 1.8 μm for each wavelength, respectively. In the measurement results, shown in Figure 12b, the reflectance can be reduced to be 0.25 when the displacement is 10.5 μm for the 786 nm wavelength. In case of the 977 nm wavelength, the SPR phenomenon can be maximized when the displacement of the piezoactuator is 9.3 μm. 

## 4. Conclusions

In this study, we verified that the Otto configuration-based SPR sensor system has the potential use as a sensor with structural flexibility, such as air-gap distance and wavelength. The surface plasmon resonance system using the air-gap interrogation method has advantages such as small size and real-time compensation of fabrication errors. The Kretschmann configuration presents flexibility limitations for reconfigurable sensor head designs. On the other hand, reconfigurable sensor design using the Otto configuration is considered to compensate for the limitation caused due to having a unique air-gap by using the air-gap interrogation method. It is expected that the results of this study can be used as background data for the development of Otto configuration-based surface plasmon resonance sensors with variable resonance characteristics, which has not been reported so far, and these features can be applied to multiple gas sensing.

## Figures and Tables

**Table 1:** Sensor characteristics of Kretschmann and Otto configurations.

Type	Kretschmann	Otto
Wavelength	Visible range	Visible and IR range
Structure	Simple	Complex
Design parameter	Refractive index, Metal thickness (<100 nm)	Refractive index, Air-gap thickness (>1 μm)
Characteristics Tunability	Low	High
Influence of Adhesion layer	High	Low

**Table 2:** Sensor characteristics for Kretschmann and Otto configurations.

Incident Angle (Degree)	41.87	42.03	42.08	42.1
FWHM of measurement (μm)	4.05	1.2	1.05	0.9
FWHM of simulation (μm)	0.7	0.25	0.05	0.15
